# Photo-responsive hydrogel-based re-programmable metamaterials

**DOI:** 10.1038/s41598-022-15453-7

**Published:** 2022-07-29

**Authors:** Herit Patel, Jiehao Chen, Yuhang Hu, Alper Erturk

**Affiliations:** 1 The George W. Woodruff School of Mechanical Engineering, Georgia Institute of Technology, Atlanta, GA 30332 USA; 2 The School of Chemical and Biomolecular Engineering, Georgia Institute of Technology, Atlanta, GA 30332 USA

**Keywords:** Gels and hydrogels, Mechanical engineering

## Abstract

This paper explores a novel programmable metamaterial using stimuli-responsive hydrogels with a demonstration of bandgap formation and tuning. Specifically, a photo-responsive hydrogel beam that can achieve re-programmable periodicity in geometric and material properties through patterned light irradiation is designed. Hydrogels consist of polymeric networks and water molecules. Many unique properties of hydrogels, including bio-compatibility, stimuli-responsiveness, and low dissipation make them ideal for enabling re-programmable metamaterials for manipulating structural dynamic response and wave propagation characteristics. Bandgap generation and tunability in photo-responsive hydrogel-based metamaterial (in the form of a diatomic phononic chain) as well as the effects of system parameters such as light exposure pattern and photo-sensitive group concentration on the bandgap width and center frequency are systematically studied. In agreement with finite-element model simulations, it is observed that an increase in light exposure region size reduces both the bandgap width and center frequency, while an increase in the concentration of photo-sensitive group increases bandgap width, attenuation and reduces its center frequency. This work unveils the potential of stimuli-response hydrogels as a new class of low-loss soft metamaterials, unlike most other soft materials that are too lossy to sustain and exploit wave phenomena.

## Introduction

It is well known that mechanical metamaterials and phononic crystals with periodic structures provide an effective way to attenuate elastic/acoustic waves over a frequency range. These engineered materials exhibiting “bandgap(s)” (frequency range in which wave propagation is forbidden) have been applied to a wide range of engineering applications targeting vibration/noise attenuation and/or wave filtering^1,2^. Metamaterials can form bandgaps via Bragg-scattering from geometric and/or material property periodicity^3,4^ or resonating (locally resonant) unit cells^5,6^. In this work, we use the word “metamaterial” in a broad sense to cover such periodic structures, including phononic crystals, i.e. engineered materials (resonant or non-resonant) that exhibit properties beyond those of ordinary materials^7^. Periodic structures made of repeating unit cells (also called lattices) readily exist in nature or can be artificially made over a wide range of length scales, from microscopic structures such as crystal lattices^8^ to multi-story buildings, bridges, etc. Research on periodic structures as simple as mass-spring chains (as a monatomic chain) can be traced back to the works of Newton and Rayleigh^9^ (for a historical account and fundamental framework, the reader is referred to Brillouin^3^). Following that, waves and bandgap formation in continuous systems have also been studied extensively^10,11^. Starting with the early 2000s, the focus shifted toward locally resonant periodic structures^5,12^. A plethora of contributions from numerous groups in the domains of elastic/acoustic metamaterials and metasurfaces have appeared in the past few decades as can be found in various review articles^1,2,13–15^.

Recently, there has been a growing interest to create metamaterials that have tunable properties by using “hard” materials, especially active materials such as piezoelectric materials^16–19^ and shape memory alloys^20,21^. For instance, shape memory alloy resonators with temperature-dependent Young's modulus^22^ were introduced to realize a temperature-controlled bandgap^20^. Thermal tuning was also investigated using metal-polymer composite metamaterial with mismatched temperature-modulus dependency^23^. Electromechanical/piezoelectric resonators have also been explored as a bandgap tuning technique in metamaterial-based structures^24–26^, including digital programming without physically altering the structure^27^. Researchers have also developed metamaterials with buckling and bistable elements for bandgap tuning^28^ and enhancement^29^. Additionally, bandgap tuning in metamaterials was designed using an inflatable structure, such that the bandgap could be controlled by gas or water pressure^30,31^. Light-responsive polymer with light controlled Young's modulus was also introduced to this field which allows researchers to use remote laser trigger to open or close bandgaps in a 3D printed resonator array^32^. Several other innovative approaches, such as tuning via magnetic field or periodic buckling in undulated beams, have also been explored^33,34^. However, the majority of bandgap tuning or programming studies have been focused on structures made from hard materials. The deformability and the reconfigurability of such structures are usually limited. Soft materials, on the other hand, provide good deformability, bio-integrability, and stimuli responsiveness. Recently, soft machines with integrated sensing, actuation, and controlling have been designed through the structural implementation of various soft active materials^35–38^. Although soft materials have been implemented into metamaterial designs, they usually serve a passive or secondary function such as creating modulus mismatch^39,40^ or reducing the buckling limit^41^. To explore the full use of soft active materials as metamaterials, the current work investigates bandgap formation and re-programming in hydrogel-based metamaterials which are orders of magnitudes softer than traditional materials and can generate dramatic shape change in response to stimuli.

Hydrogels are comprised of cross-linked polymer networks and solvents. Their good bio-compatibility^42,43^ and large deformability^44,45^ impart them with many important applications such as drug delivery^46^, wound dressing^47^, artificial organs^48^, soft sensors, actuators, and soft robots^49,50^. Many hydrogels are made stimuli-responsive that can swell or shrink in response to external stimuli such as temperature^51^, humidity^52^, pH^53^, light^54–56^, electric field^57^, and other inputs^58^. Accompanying with the volume change, hydrogels' physical properties such as density, modulus, diffusivity, and internal damping^59^ may also change. Recent studies indicate that hydrogels possess significantly less internal damping compared with traditional soft polymeric materials^60^. Compared to widely used polymeric materials such as polydimethylsiloxane (PDMS), the polyacrylamide (PAAm) hydrogel’s damping ratio is usually 4.5-5 times smaller. Furthermore, hydrogels have a close-to-water acoustic impedance, which makes them more suitable for underwater acoustics applications^61,62^. These special properties of hydrogels make them good candidates for tunable metamaterials for applications in structural dynamics and wave manipulation, especially in biomedical and underwater related areas. Among various stimuli-responsive hydrogels, photochemically-actuated hydrogels can achieve the best spatiotemporal control in tuning local morphology and properties. However, most of the conventional photo-responsive morphing hydrogels require constant light exposure to maintain their activated/deformed state^54,55^, making it impractical for dynamic applications. In efforts to address this problem, recently, we developed a new photo-responsive hydrogel by incorporating triphenylmethane leucohydroxide (TPMLH) derivatives and o-nitrobenzaldehyde (NBA) into a PAAm network. This new hydrogel exhibits decoupled light activation and swelling behavior^63^. It also exhibits large deformation/swelling while maintaining good structural integrity. By performing erasing-reprogramming operation, the hydrogel shape can be repetitively and reliably changed over many cycles without noticeable degradation. Additionally, the good spatiotemporal resolution paves the way for creating metamaterials with well-controlled and re-programmable periodic structures for generating tunable bandgaps.

This paper demonstrates bandgap formation and re-programming in a vertically suspended PAAm-co-TPMLH hydrogel beam that is locally light-activated to a periodic structure. Dynamic base excitation experiments are performed on the hydrogel cantilevers to capture bandgap formation in the motion transmissibility frequency response. A comprehensive Finite Element Method (FEM)-based model with gravitational load (and resultant static pre-deflection) is developed to predict the experiments. Additionally, we examine the variations in bandgap width and center frequency with respect to activated/non-activated segments length ratio and hydrogel parameters, such as the TPMLH concentration. Finally, the experimental data are also compared against the simple diatomic chain model for an analytical insight into the experimental trends in bandgap variations in relation to the change of mass and stiffness ratios of the unit cell.

## Results

### Overview and hydrogel metamaterial design

**Figure 1:**
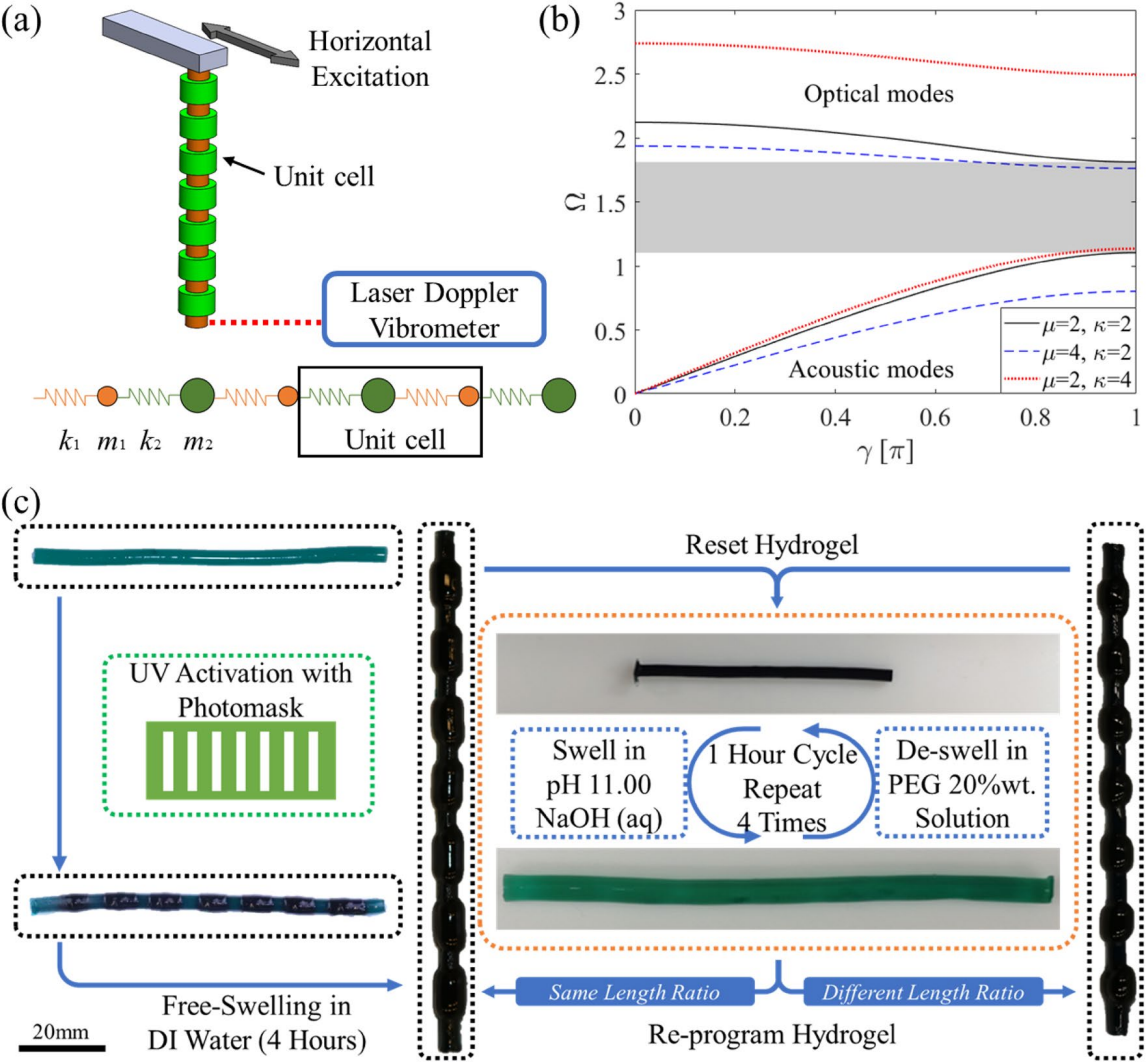
Design, modeling, and re-programming of a hydrogel periodic structure. (**a**) A continuous periodic structure and simplified diatomic chain representation for which the unit cell is defined as the combination of the repeating segments. (**b**) Dispersion plots for a diatomic chain with various unit cell mass and stiffness ratios. The shaded region in the figure represents the bandgap for *μ* = 2 and *κ* = 2 ($\Omega$ is the nondimensional frequency normalized by $\sqrt {k_{1} /m_{1} }$). (**c**) Photo-patterning and re-programming processes of the PAAm-co-TPMLH hydrogels.

Periodic repetition of geometry or material property such as Young's modulus in a structure can result in bandgaps within which the wave propagation is forbidden. In the case of a suspended periodic structure that is excited horizontally from the upper fixed end, the wave propagation response is quantified by measuring the free end motion with a Laser Doppler Vibrometer (LDV) (Fig. 1a). Because the hydrogel is soft, and the resulting cantilevered beams are slender, large excitation amplitude may generate uncontrolled off axis vibration, creating difficulties for the LDV to accurately capture the in-plane motion of the hydrogel beam tip. For this reason and also not to trigger nonlinearities, the hydrogels are only excited with low displacement amplitude for reliable data acquisition in the linear regime as well as to protect the hydrogel from damage. (Photos of the experimental setup can be found in the supplementary Fig. S1.)

The variation of the local mass and stiffness in the periodic structure controls the bandgap characteristics. The concept can be demonstrated in a diatomic chain model, which is an infinite periodic discrete chain of two different springs and masses (Fig. 1a). Here, the unit cell is defined as the periodically repeating segment of the diatomic chain. Using this model (see the supplementary document), one can derive the eigenvalues of this two-degree-of-freedom system. Dispersion curves for this simplified structure can then be created by plotting the two resultant eigenvalues versus dimensionless wavenumber, *γ* (Fig. 1b). The local minimum of the larger eigenvalue defines the upper bound of the bandgap, whereas the local maximum of the smaller eigenvalue defines the lower bound of the bandgap. Controlled variation of unit cell mass and stiffness ratio provides the foundation to tune bandgaps in periodic structures. In Fig. 1b, it can be observed that an increase in unit cell mass ratio $\mu = m_{2} /m_{1}$ results in a wider bandgap which occurs at a lower center frequency, whereas an increase in unit cell stiffness ratio $\kappa = k_{2} /k_{1}$ produces a wider bandgap at a higher center frequency.

In this work, photo-responsive hydrogels are used to generate re-programmable periodic structures. Among various photo-chemical hydrogels, our recently developed PAAm-co-TPMLH hydrogel infused with NBA solution is selected in this study for its high photo-activation efficiency and decoupled activation and swelling behavior^63^. The PAAm-co-TPMLH hydrogel is first prepared by copolymerizing TPMLH into a covalent crosslinked polyacrylamide hydrogel in dimethyl sulfoxide (DMSO)/water environment. The DMSO solvent is later removed by repeated water rinsing. To program the hydrogel, NBA is first infused into the hydrogel under basic conditions (pH 10.5). The hydrogel is then periodically activated using 365nm ultraviolet (UV) light and a pre-prepared photomask. After the photo-activation, the hydrogel is immersed in DI water for 4 hours to let it swell to its final periodic structure (Fig. 1c, black box).

Since the photo-responsiveness of the hydrogel is reversible, its unit cell periodicity can be reconfigured through an erasing-reprogramming operation. It involves washing away reaction residuals from photo-activated NBA and recovering the photodissociated triphenylmethane cation (TPM^+^) back to its non-activated neutral state. The recombination of (TPM^+^) cation and hydroxide anion is carried out in a high pH environment. For efficient removal of reaction residual and other unwanted ions in the system, the hydrogel is washed in PEG water solution and NaOH water solution alternatively and repetitively for 4 times (Fig. 1c, Orange box). Following the washing step, NBA is re-infused into the hydrogel, making it ready for another round of photo-activation and shape morphing.

### Bandgap formation in hydrogel periodic structure

**Figure 2:**
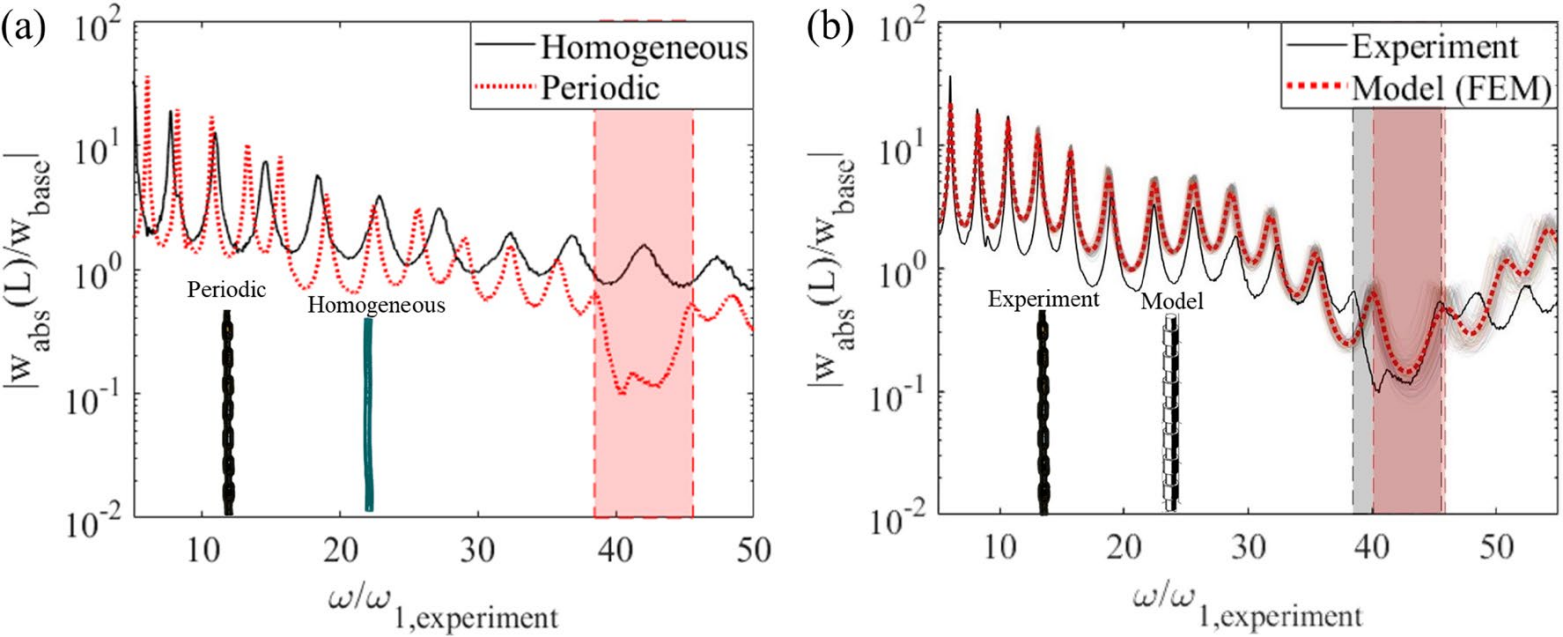
Bandgap formation in a hydrogel periodic structure. (**a**) Transmissibility FRFs for homogeneous and periodic hydrogels under base excitation. The shaded region denotes the bandgap. (**b**) Transmissibility FRF comparisons between experiment and FEM simulation for periodic hydrogel.

Figure 2a compares the tip transmissibility (absolute tip displacement per base displacement) for both a homogeneous and a periodic hydrogel structure. Unlike the homogeneous hydrogel, bandgap formation can be observed for the periodic hydrogel structure (denoted by the red patch in Fig. 2a). In this type of experiment, the bandgap is the frequency range where the magnitude of tip displacement is much less than base displacement (close to noise floor) as anticipated from the dispersion analysis due to wave attenuation.

The experimental results for the periodic structure are compared against the Finite Element Method (FEM) simulations performed in COMSOL in Fig. 2b. The FEM model performs linear frequency response analysis on a vertically suspended periodic beam which is axially deformed under gravitational load. The shaker excitation at the clamped end is emulated in the model with harmonic excitation which is applied at the top end (Fig. 1a). The model assumes the hydrogel to be a linear elastic material undergoing small deformations. Due to their low Young's moduli, the hydrogel samples tend to statically deform under their weight as they are suspended from the clamped end. The volumetric nature of gravitational loads causes larger elongation in the unit cells closer to the clamped end when compared to the free end, which in turn slightly disrupts the structural periodicity. This factor is considered in the FEM model using the gravitational body load feature which considers the uneven distribution of the static deflection along the periodic structure. Additionally, the deformation of the gel due to swelling/deswelling is associated with water diffusion which is a much slower process compared to the deformation due to vibration. Therefore, the swelling/deswelling of the gel during vibration is neglected. For this hydrogel-based periodic beam, the average diameter of the non-swollen and swollen unit cell segment is found to be 3.66 mm and 6.13 mm, respectively, while the average length for these segments is 6.68 mm and 6.69 mm, respectively. It is assumed that the entire hydrogel structure has uniform mass density, ρ = 1023.3 kg/m^3,^ and Poisson's ratio, ν = 0.49 (low compressibility due to very high-water content). The Young's modulus for the non-swollen and swollen segment is found to be 7.50±0.90 kPa and 4.72±0.82 kPa, respectively. Here, the uncertainty bounds are from the different values that are measured for each individual unit cell and multiple points on each unit of the same sample (supplementary document).

To account for the uncertainty in predicting the dynamic response of the periodic beam due to the variation in Young's modulus of each non-swollen and swollen unit, the Monte Carlo simulation method is employed^64^. The fluctuations of the modulus value are within one standard deviation of the average Young's modulus. In the simulations, a total of 150 random combinations are created to define the variations in Young's modulus of the unit cells and the responses at the tip of the beam are obtained using the FEM model. In Fig. 2b, it can be seen that the random fluctuations in Young's modulus of the unit cells result in noticeable variations in the bandgap formation. There are several combinations where the bandgap region is not as pronounced and there are combinations where bandgaps offer high attenuation. Despite these variations, one can observe an overall good agreement between the experimental results (solid black line) and model predictions (dotted red line). The mismatch between the model and the experiment is expected to be due to the transition zone between the swollen and non-swollen region is gradual and smooth in the experimental sample, while it is simulated as a sharp transition between each unit in the FEM model, as shown in Fig. 2b. Another mismatch between the model and the experimental results is the double dip inside bandgap region. Since at the bandgap region, the response amplitude is close to noise floor, it is very sensitive to experimental imperfections. The double dips and other mismatch in the bandgap region of the frequency response can be attributed to hydrogel beam imperfections (for example, the hydrogel beam may not be perfectly axisymmetric). With such defects, the hydrogel beam may develop out-of-plane motions under in-plane excitation^65.^ However, the amplitudes are very small in the bandgap region such that the double dip effect can be neglected for practical purposes.

### Bandgap formation repeatability

**Figure 3:**
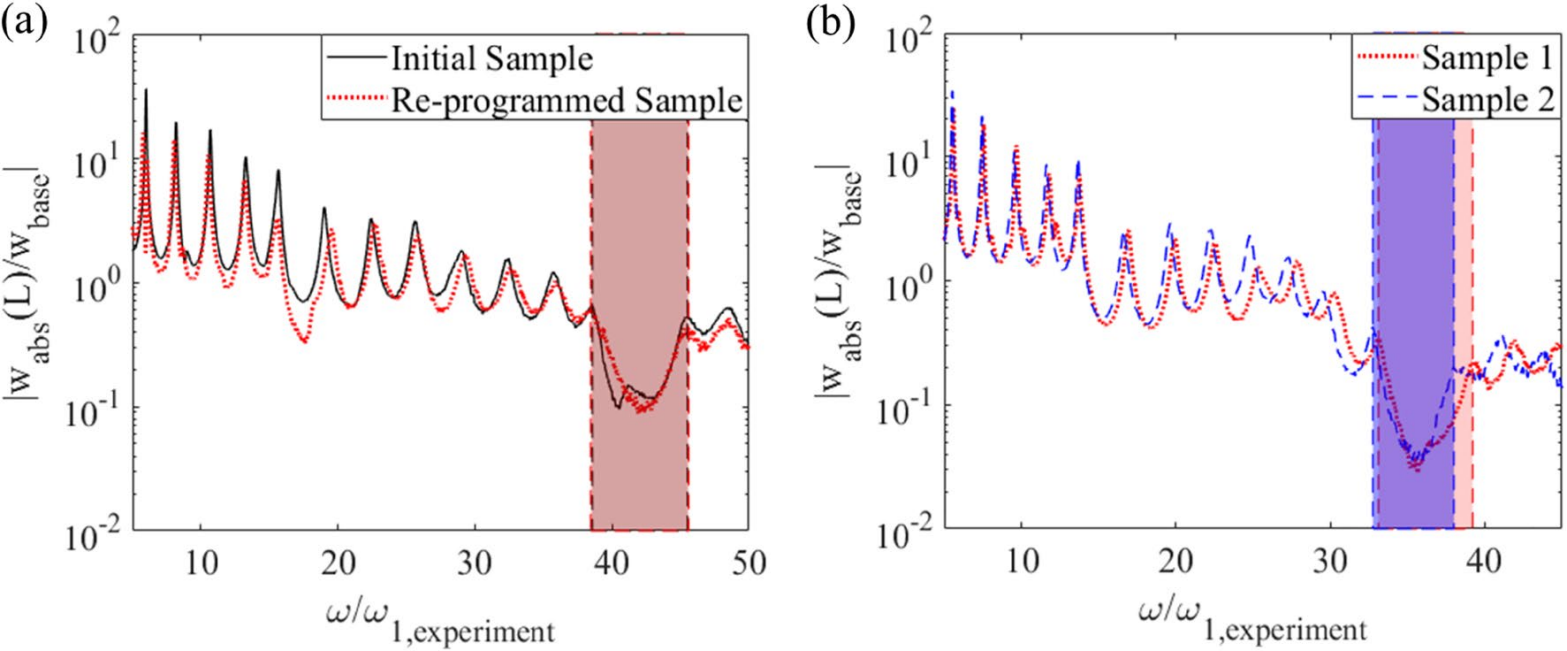
Consistency of bandgap formation. Transmissibility FRFs for (**a**) initial and re-programmed hydrogel structure with identical geometric characteristics and (**b**) two separately prepared hydrogel structures with identical geometric characteristics.

After verifying the bandgap formation of the periodically programmed hydrogel beam, we further explore the system repeatability and consistency after photo re-programming. A programmed periodic hydrogel sample with a length ratio of α = 1.26 is first reset back to its non-activated state and then re-programmed with an identical periodicity. Here, α is defined as the length ratio between the swollen region and the non-swollen region. These values are measured after the sample is photo-activated and swollen in water. Figure 3a shows the center frequencies and bandwidths of the bandgaps formed in the initial programmed periodic hydrogel beam and the re-programmed periodic beam with identical geometry. Despite the material defects and heterogeneities that commonly exist in hydrogel materials, it can be concluded that consistent bandgaps can be generated upon re-programming with the same photo pattern. Besides the bandgap repeatability of the same sample under different cycles of photo-actuation, we also explore the consistency of bandgap formation of different samples subject to the same photo-patterning. We test two separately prepared hydrogel samples that have the same chemical composition and are programmed into the same unit cell length ratio with the same photomask. Figure 3b shows the comparison of the bandgap of the two separately prepared hydrogel periodic structures. Sample 1 is reused from the previous repeatability test but re-patterned to a different unit cell length ratio of α = 1.64. Sample 2 is newly prepared with the same unit cell length ratio of α = 1.64. The slight deviation in bandgap width between the two samples is likely due to the material defects and inhomogeneity that cause the shape variations between samples after being photo-activated and swollen in water.

### Hydrogel metamaterial bandgap tuning

While the formation of the bandgap is realized and the reliability and re-programmability have been tested, we next explore the system's potential in bandgap tuning. Here, we focus on studying two control parameters: the photomask pattern that controls the length ratio of the swollen and non-swollen region, and the TPMLH concentration that controls the swelling amount of the photo-activated region. A series of samples are prepared. All the samples contain the same amount of monomer (3 mol/L acrylamide (AAm)) and crosslinker (18 mmol/L N,N'-methylenebisacrylamide (Bis)). For quantitative comparisons, we define the following relevant parameters: diameter ratio, *δ*, Young's modulus ratio, *ε*, and length ratio, *α*, of the swollen region to non-swollen region:

1 $$\delta = \frac{{d_{swollen} }}{{d_{non - swollen} }},\quad \varepsilon = \frac{{E_{swollen} }}{{E_{non - swollen} }},\quad \alpha = \frac{{L_{swollen} }}{{L_{non - swollen} }}$$

**Figure 4:**
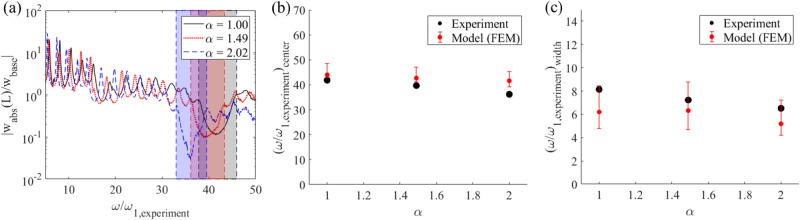
Bandgap formation in various programming scenarios of hydrogel periodic structures. (**a**) Experimental transmissibility FRFs for periodic hydrogels as α is varied from 1.00 to 2.02. (**b**) Bandgap center frequency and (**c**) width comparison between experiments and FEM simulations. The error bar is obtained through Monte Carlo analysis based on 150 random combinations for Young's modulus measured from the hydrogel sample.

Using photomasks of different window sizes, the hydrogel beam can be reconfigured to exhibit different unit cell length ratios. Since the repeatability and sample-to-sample consistency have been shown, instead of re-patterning a single sample three times, three separate samples of the same chemical composition are prepared (3 mol/L AAm, 18 mmol/L Bis, and 45 mM TPMLH) and programmed to three different unit cell length ratios. The photomasks are prepared using aluminum foils with 7 window openings cut-out at a 10 mm spacing. The widths of the window openings are 1 mm, 1.5 mm, and 2 mm, respectively. Note that the length ratio of the final swollen hydrogel is not equal to the photomask pattern ratio, mainly due to swelling of the activated segments as well as light scattering through-thickness. The tip transmissibility results of the three samples are shown in Fig. 4a. It can be observed that the bandgap shrinks, and the center frequency shifts to a lower value as the unit cell length ratio increases. Similar trends can be observed in the FEM simulation results (Fig. 4b,c respectively). The FEM model again uses the Monte Carlo method to account for random variation in Young's modulus on a unit-cell-to-unit-cell basis. For a given sample, these random combinations yield fluctuations in bandgap width and center frequency predictions. These bandgap fluctuations in the FEM model can be perceived as the effect of the difference in system sensitivity to gravitational loads with a stiffer or a softer modulus combination. A vertically hanged hydrogel will experience gravity induced non-uniform axial stretch as much as 25% at the top end. The softer the hydrogel is, the greater axial stretch non-uniformity will it experience, which in turn jeopardizes the structural periodicity, induce nonlinear material behavior (supplementary Fig. S2), and reduce the bandgap quality (yielding a narrower bandgap). For quantitative comparison, the values of the system parameters including unit cell length ratio, diameter ratio, and modulus ratio of the swollen region to the non-swollen region of each sample as well as the values of the bandgap center frequency and bandgap width are listed in Table 1. Since the samples are made with the same chemical composition, the change of photomask patterns leads to only the change of length ratio α, but not the diameter ratio δ and modulus ratio ε. The reduction in the bandgap width and attenuation can be interpreted as that the periodic beam shape converges to a homogeneous beam shape as the non-activated zone length shrinks and the unit cell length ratio increases, and a homogeneous structure is not expected to provide any bandgap. Details will be quantitatively discussed in the next section.

**Table 1:** Bandgap variation with differently programmed hydrogels of the same composition (scale bar 1 mm/div).

	TPMLH (mM)	δ	ε	Α	Bandgap center frequency (experiment)	Bandgap width (experiment)	Bandgap center frequency (model)	Bandgap width (model)
	45.0	1.65	0.67	1.00	41.9	8.1	42.8–48.6	4.8–8.4
	45.0	1.65	0.67	1.49	39.7	7.2	41.3–47.1	4.6–8.7
	45.0	1.65	0.67	2.02	36.2	6.5	39.2–45.3	4.2–7.2

**Figure 5:**
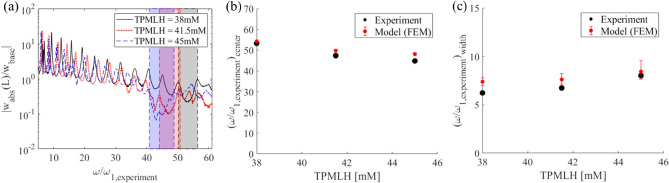
Bandgap formation in hydrogel periodic structures of different TPMLH concentrations. (**a**) Experimental transmissibility FRFs for periodic hydrogels as TPMLH concentration is varied from 38.0 to 45.0 mM. (**b**) Bandgap center frequency and (**c**) width comparison between experiments and FEM model. The error bar is obtained through Monte Carlo analysis based on 150 random combinations for Young's modulus measured from the hydrogel sample.

We next investigate the influence of TPMLH concentration on the bandgap performance of the periodic hydrogel. Here, three samples that have a TPMLH concentration of 38 mM, 41.5 mM, and 45 mM are prepared and tested. They are activated under the same photomask. The window openings on the photomask are 1.5 mm wide and 10 mm spaced from each other. The tip transmissibility results of the three samples are shown in Fig. 5a. It can be observed that the center frequency of the bandgap shifts to the left and the bandgap widens as the TPMLH concentration increases. Similar trends can be observed in the FEM simulation results (Fig. 5b,c). For quantitative comparison, the values of the system parameters including unit cell length ratio, diameter ratio, and modulus ratio of the swollen part to the non-swollen part of each sample as well as the values of the bandgap center frequency and bandgap width are listed in Table 2 . With a higher TPMLH concentration, the hydrogel swells more after photo-activation and the swollen hydrogel becomes softer. As a result, the diameter ratio δ and length ratio α increase, while the modulus ratio ε decreases as the TPMLH concentration increases. It is observed that the length ratio increase with TPMLH is not as large as the diameter ratio increase. The reason is thought to be that the light scattering near the boundary is more significant when the TPMLH concentration is low, which results in an effectively longer activated region. Consequently, it is the significant increase in diameter, a significant decrease of Young's modulus, and a moderate increase of length that leads to a wider bandgap and lower center frequency.

**Table 2:** Bandgap variation with different hydrogel compositions programmed with the same photomask. (Scale bar 1 mm/div).

	TPMLH (mM)	δ	ε	α	Bandgap center frequency (experiment)	Bandgap width (experiment)	Bandgap center frequency (model)	Bandgap width (model)
	38.0	1.36	0.90	1.31	53.3	6.2	53.9–54.5	7.0–7.8
	41.5	1.46	0.88	1.34	47.4	6.7	49.5–50.2	7.1–8.2
	45.0	1.55	0.83	1.36	44.8	8.0	47.6–48.7	7.6–9.5

### Comparisons with diatomic chain model

Based on the experimental and numerical results, an increase in unit cell length ratio (α) can shrink the bandgap and shift it to a lower center frequency, while the bandgap widens and shifts to a lower center frequency with an increase in TPMLH concentration. These trends can be interpreted using a diatomic chain model. The variations in unit cell length ratio and TPMLH concentration ultimately change the unit cell mass and stiffness ratios which are fundamentally responsible for bandgap formation in periodic structures. For a structure under bending vibration, the equivalent mass and stiffness ratios can be given by:

2 $$\mu = \delta^{2} \alpha ,\quad \kappa = \frac{{\delta^{4} }}{{\varepsilon \alpha^{3} }}$$

**Figure 6:**
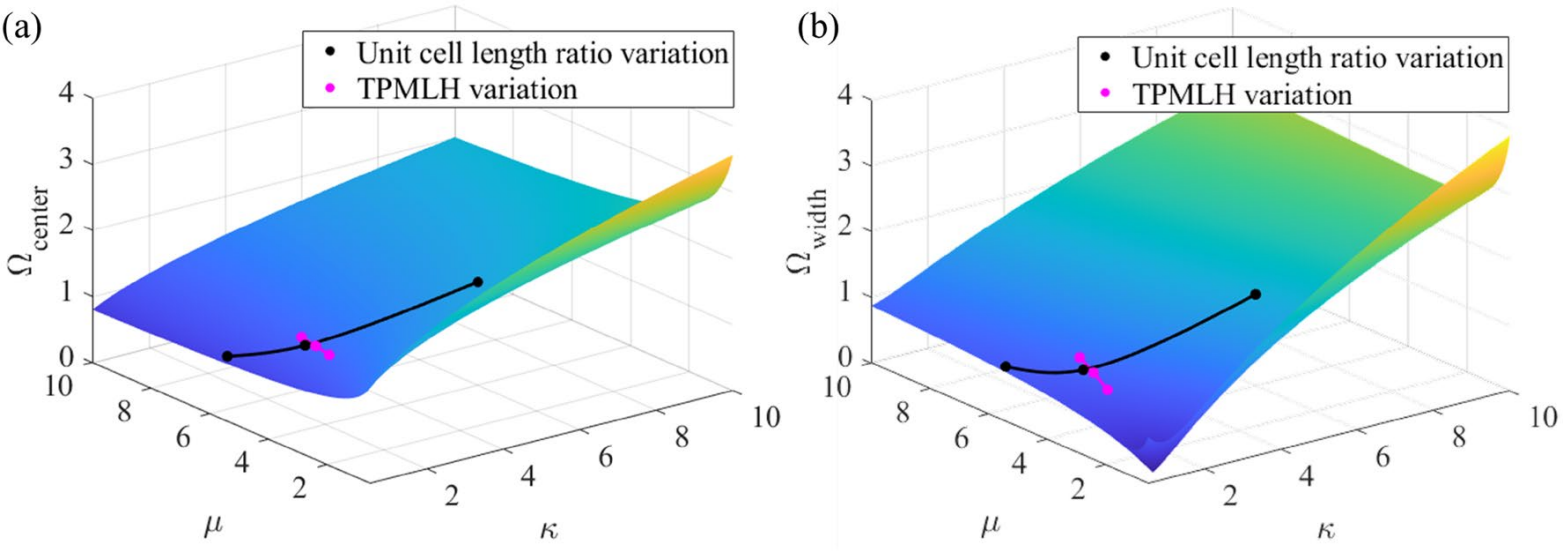
Bandgap prediction from the diatomic chain model. (**a**) Bandgap center frequency and (**b**) width variation in the diatomic chain model. The overlaid scatter points represent the bandgap widths and center frequencies for the experimental case studies as predicted by the diatomic chain model.

To analyze the experimental trends using the diatomic chain model, we use Eq. (2) to solve for the mass ratio (*μ*) and the stiffness ratio (*κ*) of the total six hydrogel samples. As shown in Fig. 6a,b, higher unit cell length ratios result in a higher mass ratio but a lower stiffness ratio which ultimately gives a narrower bandgap at a lower center frequency. On the other hand, it can be seen that an increase in TPMLH concentration results in higher mass and stiffness ratios, which ultimately gives a wider bandgap at a lower center frequency. However, for the amount of increase in mass and stiffness ratios due to TPMLH variation, the diatomic chain model predicts a negligible reduction in bandgap center frequency. The bandgap shifting trends observed with the diatomic chain model are identical to the experimental trends. Diatomic chain model is a simplification of the real structure as it neglects complex factors such as static axial deformation due to gravity load. However, the overall agreement in the trends suggests that the experimental variations in the bandgap width and center frequency are ultimately a consequence of the change in unit cell mass and stiffness ratios.

## Discussion

In this paper, we have successfully demonstrated bandgap formation and re-programming in a soft hydrogel-based periodic structure. The high-aspect-ratio hydrogels (in the form of a cantilevered beam) used in this study are the most basic scenario to demonstrate bandgap formation and re-programming in such novel materials for a fundamental understanding. Taking advantage of the photo-responsiveness and re-programmability of the PAAm-co-TPMLH hydrogel, bandgap locations can either be recreated or shifted through patterned UV exposure. The presence of the bandgap is first confirmed through base excitation experiments by measuring the response at the tip of the vertically suspended structure while it is excited at the clamped end. A comprehensive FEM-based model is developed to validate the width and center frequency of the experimental bandgaps. We further present parametric studies which allow us to control the bandgap width and location. It is observed that an increase in unit cell length ratio reduces the width and center frequency of the bandgap, whereas an increase in TPMLH concentration widens the bandgap while reducing its center frequency. Finally, the experimental trend is compared with a diatomic chain model, confirming that the variations in hydrogel structures' bandgap width and center frequency are an outcome of the change in unit cell mass and stiffness ratios. The results show the potential of hydrogel-based metamaterials and their tunability as a new class of elastic/acoustic metamaterials for applications that require low impedance, such as biomedical or underwater acoustics. Further efforts may bring a more detailed finite element model with 3D scanned hydrogel beam model and digital image correlation captured stress-stretch relation mapping to enable more accurate bandgap behavior prediction from simulation. Additionally, this work can be extended to more complex architectures for multi-directional wave propagation in 2-D and 3-D hydrogel structures.

## Materials and methods

### PAAm-co-TPMLH hydrogel synthesis

The PAAm-co-TPMLH hydrogel is synthesized by dissolving 3 mol/L monomer acrylamide (AAm) (>98% Sigma Aldrich), 18 mmol/L crosslinker *N,N*'-methylenebisacrylamide (Bis) (99% Sigma Aldrich), and 35–45 mmol/L photo-responsive agent TPMLH into a 92%vol. dimethyl sulfoxide (DMSO) (99.7% Sigma Aldrich) and 8%vol. DI water mixture. Thermo-initiator 2,2’-azobis(2-methylpropionitrile) (AIBN) (98% Sigma Aldrich) is added to initiate radical polymerization (2 mg/mL). The monomer solution is then injected into a sealed glass tube mold with an internal diameter of 2.7 mm and cured in a hot water bath at 70 ℃ for 4 h. After curing, the hydrogel rod is removed from the mold and let swell in DI water for several hours to ensure complete removal of DMSO and full saturation of water.

### Programming and erasing the hydrogel periodic structure

To program the hydrogel, the water-washed PAAm-co-TPMLH hydrogel is immersed into a 10 mmol/L NBA water solution (pH 10.5, conditioned by NaOH) to infuse the NBA molecule into the hydrogel. For speedy infusion, an additional deswelling step can be performed in advance by submerging the hydrogel in 20%wt. poly(ethylene glycol) (PEG) (Mn.10000 Sigma Aldrich) solution. After the NBA infusion, the hydrogel is cut to an 8 cm long segment and is ready for photoactivation. With the photomask placed in between the hydrogel and the 365 nm UV light (LED, 40 mW/cm^2^), the hydrogel is photoactivated into a periodic configuration. After the photo-activation, the hydrogel is immersed in DI water for 4 hours to let it swell to its final periodic geometry.

To erase the hydrogel periodicity, the PAAm-co-TPMLH hydrogel is washed in PEG water solution (Mn 10000, 20%wt.) and NaOH water solution (pH 11.0) alternatively and repetitively for four times. During this process, the hydrogel undergoes consecutive deswelling and swelling, using the in and outflow of water to effectively remove unwanted ions and other residuals inside the system. Following the washing step, NBA can be re-infused into the hydrogel, making it ready for another round of photo-activation and shape morphing.

### Dynamic testing of the hydrogel periodic structure

Base excitation tests using an electrodynamic shaker are performed using a Brüel and Kjær Vibration Exciter Type 4809. The hydrogel sample is vertically suspended with its top attached to the exciter piston via a customized acrylic clamp. A uni-axial PCB accelerometer with a signal conditioner (Kistler Type 5134) (constant gain) is used to record the acceleration at the excitation (base) end. A Polytec OFV 505 Laser Doppler Vibrometer (LDV) with a 20 kHz low pass filter is used to measure the velocity at the free (tip) end of the hydrogel to improve the signal-to-noise ratio. A 3×3mm sized reflective patch is attached to the end of the hydrogel beam to enhance laser reflection for accurate tip velocity measurements. A low voltage rectangular noise signal with Hamming window is provided to the shaker using NI signal express. The voltage signal is sent to the electrodynamic shaker via a power amplifier (LDS-PA25E) with a constant gain. The data acquisition module, NI-9223 is used to record tip and base response. Tip velocity and base acceleration are used to obtain a dimensionless tip to base displacement transmissibility frequency response function (FRF).

## Supplementary Information

Supplementary Information.

## Data Availability

All data needed to evaluate the conclusions in the paper are present in the paper and/or the Supplementary Materials.
