# Supplementary material for: Photo-responsive hydrogel-based re-programmable metamaterials
Source: Sci Rep. 2022 Jul 29;12:13033. doi: 10.1038/s41598-022-15453-7 (PMC9338311; doi:10.1038/s41598-022-15453-7)
Supplement: Supplementary file 1 — Supplementary Information. [file 41598_2022_15453_MOESM1_ESM.pdf]

Supplementary Information

Photo-responsive hydrogel-based re-programmable metamaterials

Herit Patel, Jiehao Chen, Yuhang Hu,* Alper Erturk*

Correspondence to:
yuhang.hu@me.gatech.edu (Y.H.)
alper.erturk@me.gatech.edu (A.E.)

Bloch Wave Analysis for Diatomic Chain Model

For the nth unit cell of the diatomic chain shown in Figure 1(a) (in the main text), harmonic equations of motion for the two neighboring masses can be given by:

$$(-\omega^2 m_1 + k_1 + k_2)u_{2n} - k_1(u_{2n-1} + u_{2n+1}) = 0 \quad (1)$$

$$(-\omega^2 m_2 + k_1 + k_2)u_{2n+1} - k_2(u_{2n} + u_{2n+2}) = 0 \quad (2)$$

where ω is the frequency and u is the displacement. Equations (1) and (2) can be written in matrix form for a plane wave:

$$[\mathbf{K}(\gamma) - \omega^2\mathbf{M}]\mathbf{u}(\gamma)e^{in\gamma} = 0 \quad (3)$$

in which $\gamma = \mathbf{k}L$ and $\mathbf{u}(\gamma)e^{in\gamma}$ is the spatial part of the solution as defined by the Floquet-Bloch theorem, which is commonly used to explore wave propagation in periodic structures (2,3). Here, L is the length of the unit cell (lattice parameter) and $\mathbf{k}$ is the wavevector. The stiffness matrix, **K**, and the mass matrix, **M,** can be written as:

$$\mathbf{K}(\gamma) = \begin{bmatrix} k_1 + k_2 & -k_1 e^{i\gamma} - k_2 \\ -k_1 e^{i\gamma} - k_2 & k_1 + k_2 \end{bmatrix} \quad (4)$$

$$\mathbf{M} = \begin{bmatrix} m_1 & 0 \\ 0 & m_2 \end{bmatrix} \quad (5)$$

For this two-degree-of-freedom (2-DOF) system, the eigenvalues can be found by solving for the roots of the characteristic equation:

$$\det(\mathbf{K}(\gamma) - \omega^2\mathbf{M}) = 0 \quad (6)$$

Hydrogel Image Analysis and Extracted Dimensions

In the following table, we listed the measured hydrogel dimension information, with individual segments length, diameter. Each programmed hydrogel beam contains 7 swollen segments and 8 non-swollen segments. For notation purposes, we assign numbers 1-15 to each segment in sequence. An odd number represents a non-swollen segment (Non-Activated) and an even number represents a swollen segment (Activated). The dimension of the hydrogel periodic

structure is extracted by image analysis. Table S1 listed the dimensional information for the unit cell length ratio variation experiment sample set. Table S2 listed the dimensional information for the TPMLH concentration variation experiment sample set. For simulation purposes, averaged swollen/non-swollen segment lengths are used for each sample.

Dimensional Specification for Samples with Different TPMLH Concentration

Hydrogel modulus is extracted using Neo-Hookean incompressible solid model from strain-stretch relation measured by an Instron universal tensile tester model 5944 with 50N load cell configured. Since direct measurement of the displacement during tensile testing is unreliable, image-based strain analysis is employed. Small reflective beads (0.2mm diameter) are sprinkled on the hydrogel beam surface. (Figure S2 a) The relative displacements between reflective beads can later be extracted using Tracker Software and converted to strain in each segment. Since the video-based strain measurements may contain intrinsic errors, multiple tracking points are selected to obtain a statistically significant strain value. The shear modulus is then extracted by fitting the strain-stress curve to Neo-Hookean incompressible solid model. (Figure S2 b,c) The obtained shear modulus (with 1σ envelope) as well as density are listed in Table S3 for length ratio variation set and Table S4 for TPMLH concentration variation set.

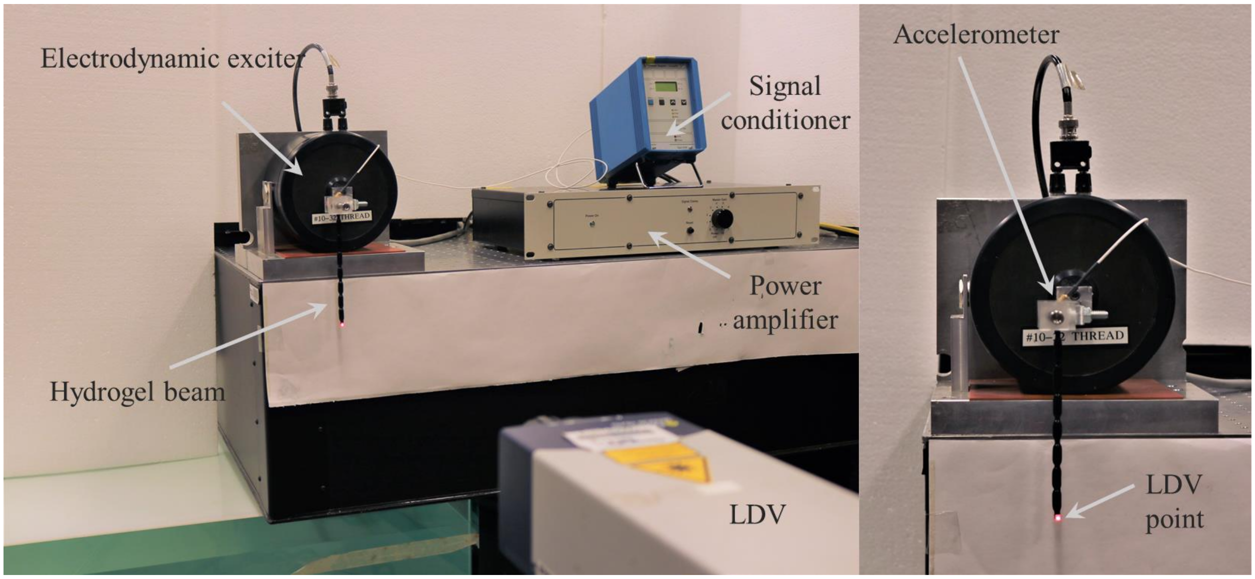

Fig. S1.
Experimental setup with the periodic hydrogel sample attached at the clamp for base excitation.

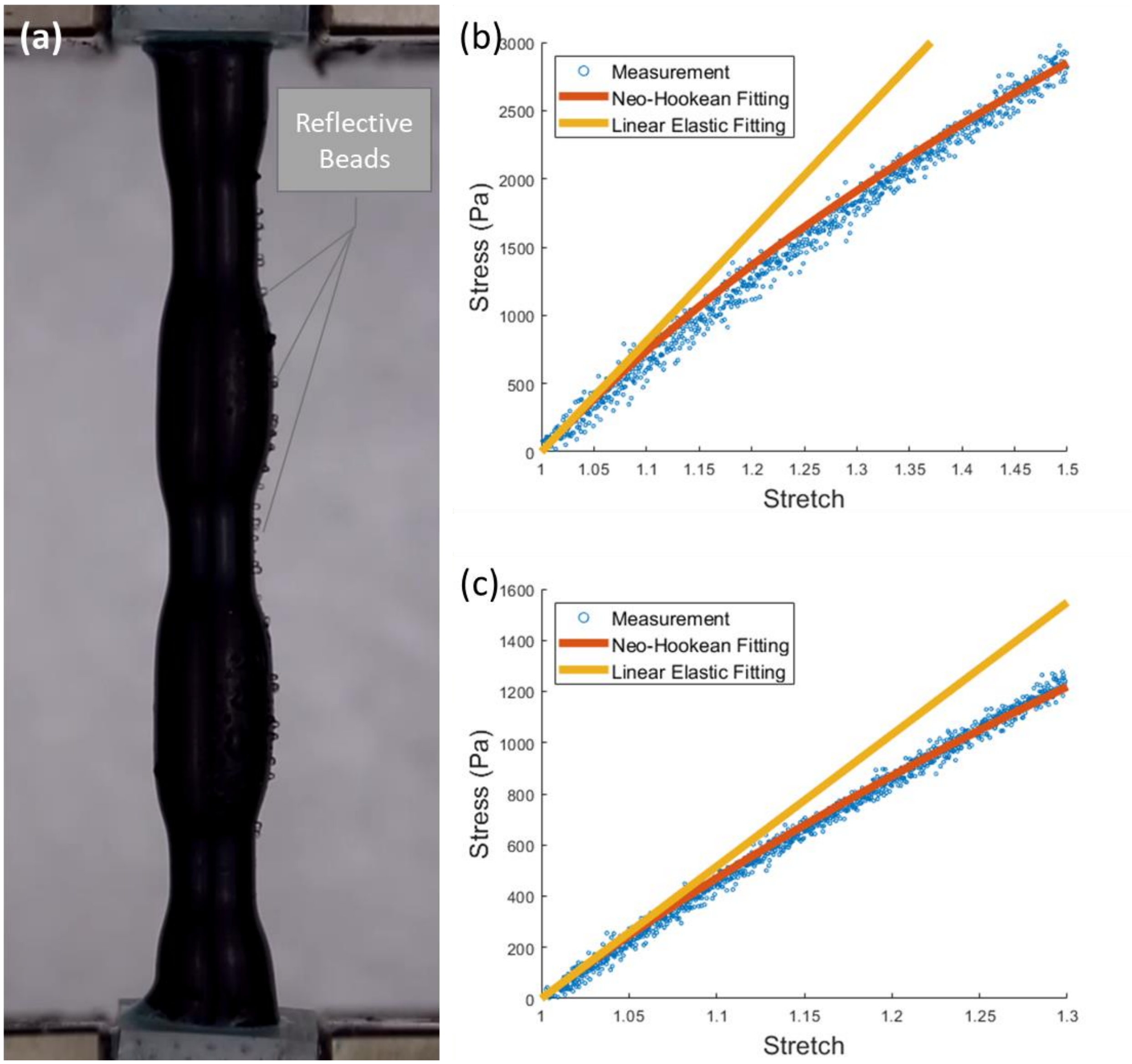

Fig. S2.

Video-based shear modulus extraction (a) Example photo of a programmed hydrogel beam with reflected beads sprinkled on its surface. (b)(c) Example comparisons of the measured strain-stretch relation, Neo-Hookean model fitting, as well as linear elastic fitting at small deformation. The top one is for non-swollen segments and the bottom one is for swollen segments.

Table S1. Dimensional specification for samples with different unit cell length ratio.

[TPMLH] = 45mM		Window Width = 1.0mm		Window Width = 1.5mm		Window Width = 2.0mm	
Position	Condition	Segment Length (mm)	Segment Diameter (mm)	Segment Length (mm)	Segment Diameter (mm)	Segment Length (mm)	Segment Diameter (mm)
1	Non-Activated	6.90	4.07	5.52	4.16	4.86	4.00
2	Activated	6.28	6.75	8.29	6.56	10.33	6.71
3	Non-Activated	6.32	3.85	5.45	3.93	4.75	4.00
4	Activated	6.64	6.51	8.33	6.62	9.85	6.73
5	Non-Activated	7.65	3.95	6.00	3.78	4.69	4.14
6	Activated	6.65	6.38	7.68	6.61	10.25	6.57
7	Non-Activated	6.82	3.87	6.40	3.77	5.30	4.08
8	Activated	6.33	6.49	7.62	6.52	9.71	6.50
9	Non-Activated	6.61	3.84	5.54	3.80	4.23	3.94
10	Activated	6.64	6.42	8.23	6.50	9.55	6.62
11	Non-Activated	6.18	3.87	4.70	3.86	5.25	3.97
12	Activated	7.39	6.40	9.28	6.47	9.72	6.46
13	Non-Activated	6.24	3.91	5.32	4.38	4.91	3.87
14	Activated	6.71	6.45	8.55	6.66	9.32	6.54
15	Non-Activated	6.82	3.99	5.46	4.07	4.85	3.94
Ave.	Non-Activated	6.69	3.92	5.55	3.97	4.85	3.99
	Activated	6.66	6.48	8.29	6.56	9.82	6.59

Table S2. Dimensional specification for samples with different TPMLH concentration.

		[TPMLH]=38.0mM		[TPMLH]=41.5mM		[TPMLH]=45.0mM	
#	Condition	Segment Length (mm)	Segment Diameter (mm)	Segment Length (mm)	Segment Diameter (mm)	Segment Length (mm)	Segment Diameter (mm)
1	Non-Activated	6.95	3.72	6.74	3.85	6.05	3.62
2	Activated	6.71	5.12	6.27	5.65	7.23	5.87
3	Non-Activated	5.07	3.69	5.49	3.81	5.05	3.77
4	Activated	6.69	5.04	6.53	5.65	7.61	5.89
5	Non-Activated	4.75	3.70	5.25	3.65	4.88	3.71
6	Activated	6.99	4.81	7.32	5.37	7.73	5.79
7	Non-Activated	4.85	3.63	5.24	3.67	5.44	3.80
8	Activated	6.81	4.99	7.84	5.45	7.14	5.62
9	Non-Activated	4.67	3.66	4.52	3.83	5.32	3.80
10	Activated	6.97	5.08	7.26	5.39	7.44	5.81
11	Non-Activated	5.09	3.78	5.07	3.74	5.35	3.84
12	Activated	6.79	5.25	7.72	5.66	7.60	5.83
13	Non-Activated	4.79	3.72	5.04	3.94	5.22	3.69
14	Activated	6.98	5.07	7.97	5.47	7.25	5.90
15	Non-Activated	5.49	3.82	6.08	3.74	6.48	3.70
Ave.	Non-Activated	5.21	3.71	5.43	3.78	5.47	3.74
	Activated	6.85	5.05	7.27	5.52	7.43	5.81

Table S3. Material property for samples used in length ratio variation experiment set

[TPMLH] = 45mM	Swollen	Non-Swollen
Shear Modulus (KPa)	3.025±0.125	3.375±0.125
Density (g/cm^3)	1.023	1.023

Table S4. Material property for samples with different TPMLH concentration

	[TPMLH]=38.0mM		[TPMLH]=41.5mM		[TPMLH]=45.0mM	
	Swollen	Non-Swollen	Swollen	Non-Swollen	Swollen	Non-Swollen
Shear Modulus (KPa)	3.025±0.125	3.375±0.125	2.725±0.125	3.100±0.100	2.575±0.225	3.100±0.150
Density (g/cm^3)	1.024	1.024	1.024	1.024	1.024	1.024
